# Understanding quality of life in bipolar disorder: associated factors and coping strategies

**DOI:** 10.3389/fpsyg.2024.1484747

**Published:** 2024-11-15

**Authors:** Hessah Alzahrani

**Affiliations:** Department of Psychology, College of Science and Humanities, Shaqra University, Shaqra, Saudi Arabia

**Keywords:** bipolar, quality of life, coping, self-blame, stress management, adaptive strategies

## Abstract

**Background:**

Bipolar disorder (BD) is a chronic mental health condition that significantly affects an individual's quality of life (QoL). While previous research has primarily concentrated on the clinical aspects of BD, there is increasing interest in understanding the factors associated with QoL in this population. This cross-sectional study aimed to assess the effects of different coping strategies on the overall QoL of individuals with BD, encompassing the physical, psychological, and social dimensions, while comparing adaptive and maladaptive coping strategies. The study sample included 96 outpatients diagnosed with BD recruited from psychiatric outpatient clinics in Riyadh and Dammam, Saudi Arabia, as well as a broader BD community. Participants completed the World Health Organization Quality of Life Brief questionnaire (WHOQOL-BREF) and Coping Orientation to Problems Experienced (Brief-COPE) questionnaires.

**Results:**

The findings indicated that problem-focused coping strategies, such as seeking support and taking direct action to manage stress, were associated with better QoL among individuals with bipolar disorder. In contrast, emotion-focused strategies such as self-blame are linked to lower QoL.

**Conclusion:**

These results contribute to the limited research on QoL in BD, particularly in Arab and Saudi societies, and underscore the need for targeted interventions aimed at developing effective coping mechanisms to enhance the wellbeing of individuals with BD.

## 1 Introduction

Bipolar disorder (BD) is a long-term condition that involves episodes of mania, hypomania, and depression and is usually accompanied by subsyndromal symptoms that occur between major episodes (Grande et al., 2016). BD is classified as a lifelong and recurrent condition that results in functional decline and decreased quality of life (Michalak et al., 2005; Bonnin et al., 2012) Given the complexity of this illness and its consequences, researchers and clinicians are not only focused on achieving clinical remission but also on functional recovery and, more recently, on overall wellbeing (Vieta and Torrent, 2016). Research on bipolar disorder has expanded to include quality of life (QOL) as an important aspect of care, in addition to symptom management and the reduction of mood symptoms. Therefore, quality of life should be considered a crucial outcome in evaluating the effectiveness of treatments (Murray and Michalak, 2012; Morton et al., 2021; Eiring et al., 2016; Haarig et al., 2016) Research has revealed that individuals with bipolar disorder often experience a lower (QOL) and impaired functioning than the general population (Çelik et al., 2022; McGinty et al., 2023; Swain et al., 2022; Islam et al., 2020). Furthermore, the quality of life of people with bipolar disorder is found to be poorer than that of people with other psychiatric disorders, including behavior disorders and non-behavior/non-mood disorders (McGinty et al., 2023; Çelik et al., 2022), suggesting that bipolar disorder has a more detrimental impact on life quality than many other mental health conditions. Despite the significant deficits in the (QOL) observed in bipolar disorder, approximately one-quarter of individuals attain good functioning, with up to 15% achieving excellent functioning (Swain et al., 2022), suggesting that a range of factors may influence (QOL). Previous studies have identified different factors that influence quality of life. These factors include personality traits, demographic characteristics, and clinical variables.

These factors include childhood trauma, social support, and psychiatric comorbidities (Rowe et al., 2023). Poor sleep management is associated with lower QoL (Vangal et al., 2024), while suicidal thoughts, cognitive reserve, and mood polarity also play significant roles (Anyayo et al., 2021; Cotrena et al., 2020; Sinha et al., 2023). While remission can enhance QoL, other important determinants include self-efficacy, mood symptoms, comorbidities, substance use (Abraham et al., 2014), and personality traits such as neuroticism and extraversion (Straten and Lodewijkx, 2006). Resilience, along with self-management and confidence, is crucial for moderating the effects of mood episodes on recovery (Echezarraga and González, 2022).

However, there is a dearth of research examining coping strategies and their impact on the quality of life of individuals with bipolar disorder. Studying Coping strategies can be crucial for enhancing the (QOL) of individuals with bipolar disorder, as these patients with bipolar disorder must cope with challenges in various aspects of their lives. Therefore, studying coping mechanisms in BD may be a promising focus for intervention.

Coping strategies can significantly influence the overall (QOL) of individuals with bipolar disorder. Studies have shown that patients with bipolar disorder often employ coping mechanisms such as secrecy and withdrawal, which are associated with higher levels of self-stigma (Au et al., 2019). Furthermore, individuals with a history of suicide attempts tend to exhibit more dysfunctional coping styles, such as behavioral disengagement and religious coping, than those without a history of suicide attempts (Poyraz et al., 2021; Cotrena et al., 2020). Currently, only a limited number of studies have investigated QoL in patients with BD, such as the Engel-Yeger et al. (2016) study, and the underlying factors associated with this construct that make it necessary still need further research despite its importance. Moreover, all existing studies have been conducted in Western societies, with insufficient data available on Eastern communities, particularly in certain cultural and regional contexts including Saudi Arabia.

In Saudi Arabia, cultural norms, family structures, societal support networks, and religious observances are integral to coping mechanisms that can substantially impact mental health and overall (QoL). The widespread stigma surrounding mental health issues in the region is prevalent among patients with mode disorders in Saudi Arabia (AlAteeq et al., 2018), potentially resulting in avoidance-based coping strategies, concealment, and reduced utilization of mental health services. This in turn may affect QoL in ways that differ from those of Western societies. Gaining insight into these culture-specific factors can aid in the development of more effective and culturally appropriate interventions for the Saudi population.

This study aimed to assess the (QOL) of individuals with bipolar disorder in Saudi Arabia and examine the relationship between coping mechanisms and quality of life, as well as how different coping strategies are associated with various aspects of quality of life in this population.

## 2 Materials and methods

### 2.1 Participants and procedures

This descriptive cross-sectional study was conducted. The study sample comprised 96 outpatients who had been diagnosed with bipolar disorder (BD) by a psychiatry specialist according to the DSM-IV (Bell, 1994) diagnosis criteria. Study participants were recruited using convenience sampling of follow-up patients at the psychiatric outpatient clinic in Eradah and the Complex Mental Health Hospital in Riyadh and Dammam between July 2023 and March 2024. The inclusion criteria for this study required participants to be at least 18 years old, have a current diagnosis of bipolar disorder (type I or II), be clinically stable (i.e., not experiencing an acute mood episode at the time of recruitment), and be able to provide informed consent. Have a single diagnosis of bipolar disorder (patients with comorbid psychiatric conditions, such as borderline personality disorder or other severe mental illnesses, were excluded). Exclusion criteria included any comorbid severe mental illness (such as schizophrenia or borderline personality disorder), current experiencing an acute mood episode, and inability to provide informed consent due to cognitive impairments or other reasons.

Only patients with a single diagnosis of bipolar disorder were included in this study. Patients with more than one psychiatric diagnosis, such as those with both bipolar disorder and borderline personality disorder, were excluded to ensure that the results focused specifically on individuals with bipolar disorder. This approach was used to minimize confounding factors associated with multiple diagnoses and to provide a clearer understanding of how bipolar disorder, in isolation, affects quality of life, and coping strategies.

### 2.2 Ethical approval

The study was conducted in accordance with the rules of research ethics on living organisms' and executive regulations issued by King Abdulaziz City for Science and Technology (KACST). This study was approved by the Committee of Research Ethics at Shaqra University Reference No. ERC_SU_S_202300006 on 19-12-2022. Informed consent was obtained from all subjects involved in the study.

### 2.3 Measures

#### 2.3.1 World Health Organization Quality of Life Brief questionnaire

The WHOQOL-BREF is a validated questionnaire designed to assess quality of life (QOL). It consists of 26 items, including one question about general QOL perception, one about overall health perception, and 24 questions that assess four specific domains of QOL: physical health (seven questions), psychological wellbeing (six questions), social relationships (three questions), and environmental factors (eight questions). Domain scores were calculated and transformed to a scale ranging from 0 to 100. These domain scores are oriented positively, meaning that higher scores indicate better quality of life. To further simplify the interpretation of the WHOQOL-BREF, participants were classified based on their transformed scores into very poor (0–20), poor (>20–40), moderate (>40–60), good (>60–80), or very good (>80–100) quality of life.

The WHOQOL-BREF was validated in an Arabic version of the WHOQOL-BREF used in current study, which was found to be both reliable and valid among Arabic-speaking individuals and in the Saudi context (Malibary et al., 2019).

#### 2.3.2 Coping Orientation to Problems Experienced

The Brief-COPE questionnaire assesses coping mechanisms through three primary strategies: Problem-Focused, Emotion-Focused, and Avoidant Coping. Problem-Focused Coping involves direct actions aimed at solving the stressor, whereas Emotion-Focused Coping manages the emotional responses associated with stress. Avoidant Coping, on the other hand, involves strategies to evade confronting a stressor directly. The tool measures these strategies on a scale from 1 to 4, with results given as average scores for each strategy as well as its subscales. A high score indicated coping strategies aimed at changing stressful situations. Arabic vision was found to be valid and reliable in a Saudi population (Alghamdi, 2020).

### 2.4 Other monitored variables

#### 2.4.1 Hospitalization history

This variable was assessed based on self-reports from the patients regarding their hospitalization history for bipolar disorder. Participants were asked to provide details about any prior hospitalization, ensuring that the information reflected their personal experiences and perspectives.

#### 2.4.2 Compliance to treatment

Compliance was assessed through patient self-reports during interviews. There was no external validation by caregivers or healthcare providers to confirm these self-reported measures. Compliance was determined by evaluating patients' adherence to prescribed medication and their attendance at scheduled follow-up appointments, relying solely on their personal accounts of treatment adherence.

#### 2.4.3 Psychotherapy enrollment

This variable refers to whether the participant had been engaged in any type of behavioral or psychological therapy provided by a psychologist, excluding pharmacological treatment. This includes therapies such as cognitive-behavioral therapy (CBT), group therapy, family therapy, or any other psychological intervention aimed at managing bipolar disorder symptoms. Participants were classified as currently undergoing therapy if they received regular sessions at the time of the study or had recently completed a therapy program.

### 2.5 Statistical analysis

The analysis was conducted using the IBM Statistical Package for the Social Sciences (SPSS), Windows 26.0 version (IBM Corporation, 2020). Sociodemographic characteristics, monitored variables, and measurement of QoL and Coping strategies scales were investigated using descriptive statistics to demonstrate the study results. Crosstabulation was used to assess the distribution of participants' QoL across different sociodemographic subgroups. Multiple linear regression analysis was used to identify any independent associations between different variables. Statistical significance was set than 0.05.

## 3 Results

As presented in Table 1, the study participants were predominantly female, with females comprising 59 of the sample (62.1%), while males accounted for 36 participants (37.9%). The age distribution within the cohort indicated a predominance of younger adults, with those aged 21–30 years representing the largest age group at 52 participants (54.7%). The age groups under 21 years and 31–40 years included 31 (32.6%) and 10 (10.5%) participants, respectively, and those over 40 years were minimally represented with only two individuals (2.1%).

**Table 1:** Socio-demographic data of participants.

**Socio-demographic data**		***N***	***N*%**
Gender	Male	36	37.9%
	Female	59	62.1%
Age	< 21	31	32.6%
	21–30	52	54.7%
	31–40	10	10.5%
	>40	2	2.1%
Social status	Single	73	76.8%
	Married	22	23.2%
Education degree	Lower degrees	2	2.1%
	High school	19	20.0%
	University	65	68.4%
	Postgraduate	9	9.5%
Medication compliance	Good	76	80.0%
	Poor	19	20.0%
Psychotherapy enrollment	Yes	58	61.1%
	No	37	38.9%
Awareness of episode triggers	Yes	77	81.1%
	No	18	18.9%
Awareness of pre-episode signs	Yes	88	92.6%
	No	7	7.4%

Social status among participants showed that a significant majority were single, including 73 individuals (76.8%), whereas married participants were fewer, totaling 22 (23.2%). In terms of educational attainment, the majority of participants held a university degree (65 participants, 68.4%), followed by high school graduates (19 participants, 20.0%), postgraduates (nine participants, 9.5%), and those with lower degrees were least represented with two participants (2.1%).

Compliance with medication was reported as good by the majority of the participants, accounting for 76 individuals (80.0%), with the remaining 19 reporting poor compliance (20.0%). Psychotherapy enrollment figures were also documented, with 58 participants engaged in psychotherapy (61.1%) and 37 not enrolled (38.9%). Awareness of episode triggers and pre-episode signs was high, with 77 (81.1%) and 88 (92.6%) participants acknowledging such awareness, respectively.

The clinical characteristics of the study participants are shown in Table 2. Previous psychosis episodes were present in 62 participants (65.3%), while 33 participants did not exhibit psychosis (34.7%). Suicidal ideation was reported by 57 participants (60.0%), while 38 participants did not report such thoughts (40.0%). Data on suicide attempts revealed that 37 participants had made at least one attempt (38.9%), whereas 58 did not attempt suicide (61.1%).

**Table 2:** Clinical characteristics of participants.

**Clinical variable**		***N***	***N*%**
Previous psychosis episode	Yes	62	65.3%
	No	33	34.7%
Suicidal thoughts	Yes	57	60.0%
	No	38	40.0%
Suicidal attempts	Yes	37	38.9%
	No	58	61.1%
No suicide attempts	No previous attempts	58	61.1%
	Once	14	14.7%
	Twice	6	6.3%
	Three times	6	6.3%
	Four times	2	2.1%
	Five times or more	7	7.4%
	Not reported	2	2.1%
Hospitalization	Never	65	68.4%
	Once	19	20.0%
	Twice	4	4.2%
	Three times	2	2.1%
	Four times	2	2.1%
	Five times or more	3	3.2%

The detailed breakdown of suicide attempts further indicates that no previous attempts were made by the majority, specifically by 58 participants (61.1%). The frequency of attempts varied, with smaller groups reporting having made one (14 participants, 14.7%), two (six participants, 6.3%), three (six participants, 6.3%), four (two participants, 2.1%), and five or more (seven participants, 7.4%) attempts. A total of 65 participants had never been hospitalized (68.4%) and 19 had been hospitalized once (20.0%), and smaller numbers were reported for two or more hospitalizations, emphasizing the chronic nature of the clinical profiles within the sample.

An examination of the quality-of-life domains, as reported in Table 3, revealed a range of scores across different dimensions assessed by the WHOQOL-Brief questionnaire. The overall WHOQOL-Brief score ranged from a minimum of 28.46 to a maximum of 88.46, with a mean score of 57.27 (±13.65 standard deviation). Specific attention to the domain of General Health showed a minimum score of 20.00 and a maximum of 100.00, averaging 57.05 (±24.57 standard deviation). The General Quality of Life scores mirrored this broad variance, with scores extending from 20.00 to 100.00 and a mean of 61.47 (±23.01 standard deviation).

**Table 3:** Descriptive statistics for quality-of-life domains.

**Domain**	**Minimum**	**Maximum**	**Mean**	**Std. deviation**
Overall WHOQOL-Brief score	28.46	88.46	57.27	±13.65
General health	20.00	100.00	57.05	±24.57
General QoL	20.00	100.00	61.47	±23.01
Physical health	28.57	88.57	57.05	±12.74
Psychological health	20.00	90.00	54.94	±15.03
Social relationships	20.00	100.00	55.71	±19.49
Environmental health	20.00	100.00	59.13	±17.12

Analysis of the Physical Health domain showed scores fluctuating between 28.57 and 88.57, with an average value of 57.05 (±12.74 standard deviation). Psychological Health scores were similarly distributed, ranging from 20.00 to 90.00, with an average of 54.94 (±15.03 standard deviation). Social Relationships were evaluated with scores spanning from 20.00 to 100.00, resulting in an average score of 55.71 (±19.49 standard deviation). Lastly, the Environmental Health domain displayed scores from 20.00 to 100.00, with an average score of 59.13 (±17.12 standard deviation).

**Figure 1:**
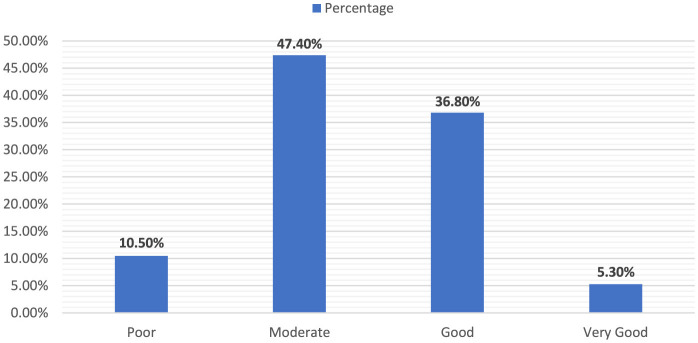
WHOQOL-Brief score classification.

The classification of the WHOQOL-Brief scores into different qualitative categories in Figure 1 reflects the diverse perceptions of quality of life in the study cohort. The sample illustrated a broad range of QOL, with a significant majority reporting moderate to good levels, pointing to a generally positive but varied assessment of life quality among the participants.

Furthermore, a minority of participants were categorized as having a poor quality of life, consisting of 10 individuals (10.5%). The largest segment of the cohort was classified under “Moderate” quality of life, encompassing 45 participants (47.4%). The “good” category included 35 individuals (36.8%), and a small fraction was deemed to have a Very Good quality of life, including only five participants (5.3%).

As illustrated in Table 4, the association between (QoL) and sociodemographic data has varied outcomes. Gender distribution showed that males were represented in the 'Poor' QoL category by 4 individuals (4.2%), “Moderate” by 17 (17.9%), “Good” by 11 (11.6%), and “Very Good” by another 4 (4.2%). Females were slightly more likely to fall into the “Good” category with 24 participants (25.3%), while 28 were “Moderate” (29.5%), 6 “Poor” (6.3%), and only 1 “Very Good” (1.1%).

**Table 4:** Association between quality of life and socio-demographic data.

**Socio-demographic data**		**WHOQOL-class**				***p*-value**
		**Poor**	**Moderate**	**Good**	**Very good**	
Gender	Male	4	17	11	4	0.363
	Female	6	28	24	1	
Age	< 20	3	15	13	0	0.518
	21–30	5	25	18	4	
	31–40	1	5	3	1	
	>40	1	0	1	0	
Social status	Single	6	32	31	4	0.033^*^
	Married	4	13	4	1	
Education degree	Lower degrees	1	0	1	0	0.142
	High school	2	10	7	0	
	University	7	31	22	5	
	Postgraduate	0	4	5	0	
Compliance	Good	4	35	32	5	0.0001^*^
	Poor	6	10	3	0	
Psychotherapy	Yes	4	27	23	4	0.073
	No	6	18	12	1	
Triggers awareness	Yes	7	35	31	4	0.135
	No	3	10	4	1	
Signs of attack	Yes	9	41	34	4	0.485
	No	1	4	1	1	

*p < 0.05 indicates statistical significance.

Age-wise distribution revealed that participants under 20 years had a more even distribution across “Poor” to “Good” QoL categories, but none in “Very Good.” Those aged 21–30 were more represented in the “Moderate” and “Good” categories. Participants aged 31–40 and over 40 had minimal representation, especially in the higher QoL categories.

Single participants showed a significant spread across all categories except “Very Good,” with 6 (6.3%) in “Poor,” 32 (33.7%) in “Moderate,” and 31 (32.6%) in “Good.” Married participants were less likely to report “Good” QoL, with only 4 (4.2%) in this category. Regarding education, participants with university degrees showed higher occurrences in “Moderate” and “Good” categories. In contrast, those with high school or lower qualifications were less represented in higher QoL categories.

Compliance with medication was strongly associated with better QoL outcomes, with good compliance being linked to better QoL scores across 35 participants (36.8%). Poor compliance correlated with higher presence in the “Poor” QoL category. The data regarding psychotherapy enrollment did not show strong variations across QoL categories, indicating a mixed impact on QoL outcomes. Similarly, awareness of episode triggers and pre-episode signs showed varied distributions across QoL categories, with no clear pattern emerging from the data.

As presented in Table 5, the clinical characteristics of the participants indicated different effects on their quality of life. Previous psychosis episode was present in 62 participants (65.3%), and this group showed a higher presence in “Moderate” and “Good” QoL categories, whereas those without psychosis had a slightly more even distribution across QoL categories. Suicidal thoughts were reported by 57 participants (60.0%), with a notable concentration in the “Moderate” and “Good” quality of life (QoL) categories. This finding highlights an intriguing pattern where individuals experiencing suicidal thoughts still reported moderate to good QoL scores, with 28 participants categorized as “moderate” and 26 as “good.” Conversely, participants without suicidal thoughts displayed a somewhat similar distribution, suggesting that both groups reported comparable levels of QoL. Specifically, those without suicidal thoughts showed an even distribution across the “Moderate” and “Good” categories. This similarity indicates that the presence of suicidal thoughts does not necessarily correlate with a significantly lower perception of QoL, emphasizing the complexity of how suicidal ideation interacts with overall wellbeing.

**Table 5:** Association between quality of life and clinical variables.

**Clinical variable**		**WHOQOL-class**				***p*-value**
		**Poor**	**Moderate**	**Good**	**Very good**	
Previous psychosis episode	Yes	7	28	26	1	0.349
	No	3	17	9	4	
	No	0	17	17	4	
Suicidal attempts	Yes	6	16	15	0	0.122
	No	4	29	20	5	
No suicide attempts	No previous attempts	4	29	20	5	0.057
	Once	1	6	7	0	
	Twice	1	2	3	0	
	Three times	1	5	0	0	
	Four times	0	1	1	0	
	Five times or more	2	2	3	0	
	Not reported	1	0	1	0	
Hospitalization	Never	10	29	23	3	0.010^*^
	Once	0	12	7	0	
	Twice	0	2	1	1	
	Three times	0	0	2	0	
	Four times	0	2	0	0	
	Five times or more	0	0	2	1	

*p < 0.05 indicates statistical significance.

Suicidal attempts showed a significant representation in the “Moderate” QoL category. The number of attempts did not significantly vary the distribution in QoL categories, except for those with more attempts, which were generally more represented in the lower QoL categories. Hospitalization history revealed a strong link between never being hospitalized and higher QoL scores, with 29 participants (30.5%) in “Moderate” and 23 (24.2%) in “Good.” Those hospitalized once or more showed a significant drop in “Good” QoL scores, emphasizing the potential impact of hospitalization on perceived QoL.

The analysis of the coping strategies employed by participants delineated three main types: Problem-focused, Emotion-focused, and Avoidant coping. The Problem-focused strategy, which involves direct actions aimed at mitigating the stressor, showed a minimum score of 1.12 and a maximum of 4.00, with an average score of 2.68 (±0.64 standard deviation). The data in Table 6 indicate relatively high utilization of this strategy, with some variability among the participants.

**Table 6:** Descriptive statistics for coping strategies.

**Coping strategy**	**Minimum**	**Maximum**	**Mean**	**Std. deviation**
Problem-focused	1.12	4.00	2.68	±0.64
Emotion-focused	1.00	4.00	2.71	±0.52
Avoidant coping	1.25	4.00	2.43	±0.51

The Emotion-focused coping strategy, which manages emotional responses to stress, was observed with a minimum score of 1.00, and a maximum of 4.00. It had a slightly higher average score of 2.71 (±0.52 standard deviation), suggesting that managing emotional responses to stress was slightly more prevalent or effective among the study participants. Nonetheless, Avoidant coping, characterized by evasion of the stressor, recorded scores ranging from 1.25 to 4.00, with a lower average of 2.43 (±0.51 standard deviation). This lower mean suggests that while still employed, avoidant coping is less favored than Problem-focused and Emotion-focused strategies among the participants.

The Problem-focused method, which involves direct actions to reduce the stressor, showed scores ranging from 1.12 to 4.00, with an average of 2.68 (±0.64 standard deviation). This method includes strategies such as actively solving problems, seeking helpful information, making plans, and seeing the positive aspects of situations. Higher scores indicate that participants are actively trying to change their stressful situations, showing strong problem-solving skills, and a practical approach to challenges. The data suggest that this method is quite popular among participants, with minimal variation in their score differences.

The Emotion-focused coping method, which deals with the emotional side of stress, had scores from 1.00 to 4.00, with a slightly higher average of 2.71 (±0.52 standard deviation). This includes strategies such as talking about one's feelings, getting emotional support from others, using humor, accepting the situation, blaming oneself, and turning to religion. This suggests that managing emotions is a slightly more common or effective approach among the participants.

On the other hand, Avoidant coping, which involves avoiding the stressor through tactics like distracting oneself, denying the problem, using substances, or giving up on certain actions, had scores between 1.25 and 4.00, with an average of 2.43 (±0.51 standard deviation). The lower average score suggests that avoidant coping is less popular than problem- and emotion-focused strategies among participants. Generally, lower scores in this area are considered more adaptive, as relying heavily on avoidant coping might not be an effective way to handle stress.

As illustrated in Table 7, the study evaluated the average scores for different coping strategy subscales on a scale from 1 to 4. Among problem-focused strategies, active coping had the highest mean score of 2.8316 (Std. Deviation = 0.827), followed by planning with a mean of 2.7421 (Std. Deviation = 0.850). The use of informational support and positive reframing had mean scores of 2.4632 (Std. Deviation = 0.940) and 2.7053 (Std. Deviation = 0.929), respectively.

**Table 7:** Average score for coping strategies subscales.

**Coping strategy**	**Subscale**	**Mean (1–4 score)**	**Std. deviation**
Problem-focused	Active coping	2.8316	0.827
	Use of informational support	2.4632	0.940
	Positive reframing	2.7053	0.929
	Planning	2.7421	0.850
Emotion-focused	Emotional support	2.5895	0.913
	Venting	2.6789	0.868
	Humor	2.4263	1.010
	Acceptance	2.9263	0.847
	Self-blame	2.8842	0.977
	Religion	2.7737	1.068
Avoidant coping	Self-distraction	2.8474	0.863
	Denial	2.0632	0.995
	Substance use	2.6053	0.754
	Behavioral disengagement	2.0947	0.920

For emotion-focused strategies, acceptance had the highest average score of 2.9263 (Std. Deviation = 0.847). Self-blame followed closely with a mean score of 2.8842 (Std. Deviation = 0.977). Religion, Emotional support, and venting had a mean score of 2.7737 (Std. Deviation = 1.068), and 2.5895 (Std. Deviation = 0.913), and 2.6789 (Std. Deviation = 0.868), respectively. Humor had the lowest mean score within this category, at 2.4263 (Std. Deviation = 1.010), respectively.

Among avoidant coping strategies, self-distraction had the highest mean score of 2.8474 (Std. Deviation = 0.863), followed by substance use, with a mean of 2.6053 (Std. Deviation = 0.754). Behavioral disengagement and denial had mean scores of 2.0947 (Std. Deviation = 0.920) and 2.0632 (Std. Deviation = 0.995), respectively.

Furthermore, the linear regression analysis to predict Quality of Life (QOL) based on sociodemographic and clinical variables included variables such as Gender, Age, Social status, education degree, compliance, psychotherapy, trigger awareness, signs of attack, previous psychosis episode, suicidal thoughts, suicide attempts, Number of Suicide attempts, and hospitalization.

Among the variables, suicidal thoughts showed a significant positive effect on QOL, with an unstandardized coefficient (B) of 0.378 and a standardized coefficient (Beta) of 0.250. The effectiveness of this variable was further supported by its *t*-value of 2.360 and a significance level of *p* = 0.021, suggesting that it is a significant predictor of QOL changes.

Hospitalization also demonstrated a significant positive effect on QOL, with an unstandardized coefficient (B) of 0.108 and a standardized coefficient (Beta) of 0.228. The *t*-value of 2.274 and significance level of *p* = 0.026 underscore its importance as a predictor of QOL.

In contrast, other variables such as Gender, Age, Social status, education degree, compliance, psychotherapy, trigger awareness, signs of attack, previous psychosis episode, suicidal attempts, and Number of Suicide attempts did not show statistically significant effects on QOL. For instance, social status had a negative effect with a B of −0.338 and a beta of −0.192, but this was not significant (*t* = −1.781, *p* = 0.079). Similarly, Compliance showed a negative effect (B = −0.354, Beta = −0.191), but this was not significant (*t* = −1.778, *p* = 0.079).

Overall, the analysis indicated that suicidal thoughts and hospitalization were significant predictors of QOL, whereas other sociodemographic and clinical variables did not show significant effects in predicting QOL changes. As reported in Table 8.

**Table 8:** Regression analysis of QOL based on sociodemographic and clinical variables.

**Model**	**B**	**Standard error**	**Beta**	***t***	**Significance**
Gender	−0.191	0.161	−0.125	−1.187	0.239
Age	0.067	0.115	0.063	0.587	0.559
Social status	−0.338	0.190	−0.192	−1.781	0.079
Education degree	0.105	0.129	0.085	0.813	0.419
Compliance	−0.354	0.199	−0.191	−1.778	0.079
Psychotherapy	−0.177	0.151	−0.117	−1.174	0.244
Triggers awareness	−0.135	0.205	−0.071	−0.656	0.514
Signs of attack	−0.298	0.307	−0.105	−0.970	0.335
Previous psychosis episode	0.232	0.160	0.149	1.453	0.150
Suicidal thoughts	0.378	0.160	0.250	2.360	0.021
Suicidal attempts	0.086	0.215	0.056	0.398	0.691
No suicide attempts	−0.010	0.055	−0.028	−0.185	0.854
Hospitalization	0.108	0.048	0.228	2.274	0.026

Linear regression analysis was conducted to predict Quality of Life (QOL) based on three coping strategies: Problem-focused, Emotion-focused, and Avoidant coping. The model produced significant results, *F*_(3, 91)_ = 11.313, *p* < 0.001, with an *R*^2^ of 0.272. This indicates that ~27.2% of the variance in QOL is explained by these coping strategies, as modeled. Overall, the analysis highlighted problem-focused coping as a significant predictor of better QOL, whereas Emotion-focused and Avoidant coping did not significantly contribute to predicting QOL changes. Further details are provided in Table 9.

**Table 9:** Regression analysis of QOL based on coping strategies.

**Model**	**B**	**Standard error**	**Beta**	***t***	**Significance**
Problem-focused	2.890	0.167	0.594	5.244	0.000
Emotion-focused	−0.215	0.177	−0.151	−1.216	0.227
Avoidant coping	−0.077	1.46	−0.053	−0.529	0.598

The adjusted *R*^2^ value was 0.248, reflecting a slightly more conservative estimate that accounted for the number of predictors used in the model. Among the strategies, problem-focused coping showed a significant positive effect on QOL, with an unstandardized coefficient (B) of 2.890 and a standardized coefficient (beta) of 0.594, indicating a strong positive influence. The effectiveness of this strategy was further underscored by its *t*-value of 5.244 and a significance level of *p* < 0.001, suggesting that it is a highly effective means of improving QOL.

In contrast, emotion-focused coping had a negative effect, although not statistically significant, with a B of −0.215 and a Beta of −0.151, with a *t*-value of −1.216 and *p* = 0.227. Similarly, Avoidant coping also showed a negative association with QOL (B = −0.077, Beta = −0.053), but this was also not significant (*t* = −0.529, *p* = 0.598), indicating that these strategies might not be effective or could potentially detract from QOL.

As illustrated in Table 10, a regression analysis was conducted to predict Quality of Life (QOL) based on coping strategy subscales. Among problem-focused strategies, active coping showed a significant positive effect on QOL, with an unstandardized coefficient (B) of 0.340 and a standardized coefficient (Beta) of 0.378. This was further supported by its *t*-value of 3.685 and a significance level of *p* =0.000. Similarly, the Use of informational support also demonstrated a significant positive effect, with a B of 0.271 and beta of 0.342, supported by a *t*-value of 2.706 and a significance level of *p* = 0.008. Positive reframing and planning did not have significant effects on QOL, with *p*-values of 0.536 and 0.465.

**Table 10:** Regression analysis of QOL based on coping strategies subscales.

**Coping strategy**	**Subscale**	**B**	**Standard error**	**Beta**	***t***	**Significance**
Problem-focused	Active coping	0.340	0.092	0.378	3.685	0.000
	Use of informational support	0.271	0.100	0.342	2.706	0.008
	Positive reframing	0.055	0.089	0.069	0.621	0.536
	Planning	−0.067	0.091	−0.077	−0.734	0.465
Emotion-focused	Emotional support	−0.225	0.114	−0.276	−1.985	0.051
	Venting	−0.011	0.086	−0.013	−0.128	0.898
	Humor	−0.004	0.072	−0.006	−0.059	0.953
	Acceptance	0.149	0.106	0.169	1.405	0.164
	Self-blame	−0.255	0.067	−0.335	−3.815	0.000
	Religion	0.013	0.073	0.019	0.181	0.856
Avoidant coping	Self-distraction	0.068	0.089	0.079	0.764	0.447
	Denial	0.004	0.072	0.005	0.056	0.955
	Substance use	−0.079	0.110	−0.080	−0.723	0.472
	Behavioral disengagement	0.005	0.084	0.006	0.061	0.951

In the Emotion-focused category, emotional support had a negative effect on QOL, which was marginally significant, with a B of −0.225, Beta of −0.276, *t*-value of −1.985, and a *p*-value of 0.051. Self-blame also showed a significant negative effect on QOL, with a B of −0.255, beta of −0.335, *t*-value of −3.815, and a significance level of *p* = 0.000. Other subscales in this category, such as Venting, Humor, Acceptance, and Religion, did not show significant effects on QOL, with *p*-values ranging from 0.164 to 0.953.

For avoidant coping strategies, none of the subscales, including Self-distraction, Denial, Substance use, and behavioral disengagement, showed significant effects on QOL. Their *p*-values ranged from 0.447 to 0.955, indicating no significant predictive value for QOL changes.

Overall, Table 10 highlights that within problem-focused coping strategies, active coping and the use of informational support are significant positive predictors of QOL. In the Emotion-focused category, self-blame was a significant negative predictor of the QOL. Other subscales across categories did not show a significant predictive value for QOL changes.

## 4 Discussion

The present study examined the relationship between various factors and quality of life (QoL) in individuals with bipolar disorder. The findings suggest that while gender, age, and history of psychosis are not significantly associated with overall QoL, other factors, such as medication adherence, hospitalization, and coping strategies, play a crucial role.

The current research indicates that among the participants, both sexes showed similar distributions across various QoL categories, and no significant differences were observed based on sex. While previous studies have reported different results, suggesting that females may experience lower QoL than males (Shokrgozar et al., 2024; de la Cruz et al., 2013), these discrepancies might be attributed to factors such as medical comorbidities, as noted in de la Cruz et al. (2013).

In addition, while the younger participants in this study generally reported better QoL, age alone was not a significant factor when considering other factors. This finding is consistent with (Parikh and Panse, 2019), who highlighted the negative impact of the duration of illness and the total number of episodes on QoL in older patients with bipolar disorder. This suggests that the cumulative effects of the disorder over time, rather than age, contribute to a decline in QoL.

Regarding medication adherence and QoL, this study underscores the strong association between medication adherence and improved QoL. This aligns with, previous research demonstrating that consistent medication use is crucial for managing bipolar disorder symptoms and enhancing overall wellbeing (Mishra et al., 2017; Okazaki et al., 2023). Hospitalization was positively associated with quality of life (QoL), likely due to the intensive treatment and support provided. This finding aligns with the literature, which indicates that hospitalization is a critical intervention for patients with bipolar disorder, particularly during acute episodes (Nierenberg et al., 2023). It offers a structured environment for comprehensive care, including medication management and therapeutic interventions, which effectively stabilizes mood and reduces symptoms, thereby enhancing overall QoL.

While a history of psychosis was not significantly associated with quality of life (QoL) in the current study, individuals who had experienced psychosis reported moderate to good QoL. This suggests that although psychotic symptoms can complicate the severity of bipolar disorder, they do not necessarily lead to uniformly poor QoL outcomes. This finding is supported by a study by Chakrabarti and Singh (2022), who conducted a systematic review of psychotic symptoms in bipolar disorder. Their review highlighted that psychotic symptoms are common in bipolar disorder but do not always negatively affect the course or outcomes of the illness. Many patients with a history of psychosis can achieve significant functional recovery and may maintain a satisfactory QoL. This review suggests that factors, such as resilience and supportive environments, can mitigate the impact of these symptoms. Thus, the presence of psychotic symptoms does not automatically lead to diminished QoL, indicating that various factors contribute to the overall wellbeing of this population.

### 4.1 Coping strategies and quality of life in bipolar disorder

Among problem-focused strategies, active coping, defined as taking direct action to address stressors through problem-solving, was found to have a significant positive association with both quality of life (QoL) and the use of informational support. This finding is consistent with those of Boostani and Tabatabaeinejad (2023) and Karbasi et al. (2023), who highlighted that active problem solving and seeking help from others enhance mental wellbeing and stress management, thereby improving QoL in individuals with bipolar disorder. However, strategies such as positive reframing and planning did not show significant effects on QoL, emphasizing that direct action and support are more effective in managing stress than planning or trying to maintain a positive outlook.

Problem-focused coping strategies aim to address the root causes of stressors rather than alleviate symptoms or manage emotional responses. Previous studies suggest that engaging in these strategies can lead to improvements in mental wellbeing, which are critical aspects of QoL in individuals with bipolar disorder. For instance, Elshaer (2023) and Budimir et al. (2021) found that during the COVID-19 pandemic, active coping strategies significantly enhanced mental health and QoL, illustrating the broader applicability of these findings beyond bipolar disorder.

The positive association between active coping, informational support, and improved QoL among patients with BD underscores the importance of incorporating these elements into treatment strategies. Interventions aimed at strengthening active coping skills and providing adequate informational support may enhance patient QoL outcomes. This approach could complement pharmacological treatment and contribute to a more holistic care model.

In the emotion-focused category, self-blame emerged as a negative factor affecting QoL in individuals with bipolar disorder. This aligns with the findings of Sinead et al. (2022), Guštin et al. (2022), and Jaeckle et al. (2021), who noted that self-blame was linked to self-criticism and feelings of worthlessness, exacerbating anxiety and depression. Research indicates that self-blame correlates with increased rates of anxiety and depression, which are common comorbidities in bipolar disorder, where mood fluctuations and emotional regulation challenges are prevalent. Thus, addressing self-blame through therapeutic interventions could be vital for improving QoL in patients with BD.

Interestingly, while emotional support is generally seen as beneficial, Granek et al. (2018) study suggested that excessive reliance on emotional coping strategies by partners of bipolar patients may lead to strain and negatively affect their QoL. This indicates that, while emotional support is essential, an overemphasis on emotional coping without sufficient professional and psychosocial support may not be enough to maintain a high QoL for both bipolar patients and their partners. Further research is needed to clarify the specific effects of emotion-focused coping strategies on the QoL of patients with BD and to develop targeted interventions that promote adaptive coping mechanisms.

## 5 Strengths and limitations of the study

This study addresses a significant gap in the existing literature concerning bipolar disorder and quality of life (QoL) within the Saudi Arabian context. This study contributes to a more nuanced understanding of disorder in diverse cultural settings. A comprehensive evaluation of QoL, coping strategies, and relevant demographic and clinical variables resulted in a robust dataset that provided valuable insights. The use of validated measures such as the WHOQOL-BREF and Brief-COPE ensured the reliability and validity of the collected data. Additionally, a clear methodology that outlines detailed inclusion and exclusion criteria, data collection procedures, and statistical analyses enhances both transparency and replicability. Although this study offers insights into factors associated with quality of life (QoL) and coping strategies among individuals with bipolar disorder, it has some limitations. The cross-sectional design limits causal inference, necessitating longitudinal studies to examine the temporal relationships. Although self-reported data on treatment compliance were collected, systematic screening for somatic comorbidities, which could affect QoL and coping strategies, was not conducted. Reliance on self-reported data may introduce bias, suggesting the need for more objective evaluations such as medical record reviews or standardized assessments. Despite controlling for several demographic and clinical variables, other factors affecting QoL were not analyzed, warranting further research to explore these unmeasured variables for a comprehensive understanding of QoL factors in bipolar disorder. The cultural context of Saudi Arabia raises questions regarding the generalizability of these results to other populations. Excluding participants with comorbid psychiatric conditions may limit their applicability to more complex cases of bipolar disorder. Additionally, disorder-specific tools were not used to capture unique QoL aspects because of the lack of validated Arabic versions of measures, such as the Bipolar Quality of Life Scale (BQOL) at the time. Future research should explore the adaptation and validation of disorder-specific QoL measures, such as the BQOL, in Arabic-speaking populations to address this gap.

## 6 Conclusions

This study offers valuable insights into bipolar disorder (BD) and its impact on quality of life (QoL). These findings suggest that medication adherence and hospitalization are associated with better QoL outcomes in individuals with BD. Furthermore, the study highlights the importance of problem-focused coping strategies, which are linked to enhanced QoL, while emotion-focused strategies, such as self-blame, are associated with lower QoL. This emphasizes the need to incorporate active coping skills and informational support into treatment plans to improve patient outcomes related to QoL. Additionally, further research is warranted to examine the role of emotion-focused coping strategies in QoL and to develop interventions that encourage adaptive coping mechanisms. However, it is important to note that the cross-sectional design of the study limits its ability to establish causality; thus, the findings should be interpreted as associations rather than definitive cause-and-effect relationships.

## Data Availability

The data that support the findings of this study are available upon reasonable request, requests to access the datasets should be directed to halzahrani@su.edu.sa. However, due to ethical considerations, the data can only be shared for research purposes and cannot be used in other studies without prior consent from the participants.
