# Corrigendum: Understanding quality of life in bipolar disorder: associated factors and coping strategies

**DOI:** 10.3389/fpsyg.2025.1571072

**Published:** 2025-02-19

**Authors:** Hessah Alzahrani

**Affiliations:** Department of Psychology, College of Science and Humanities, Shaqra University, Shaqra, Saudi Arabia

**Keywords:** bipolar, quality of life, coping, self-blame, stress management, adaptive strategies

In the published article, there was an error in affiliation. Instead of “Shagra University,” it should be “Shaqra University.” The correct full affiliation is shown below.

“Department of Psychology, College of Science and Humanities, Shaqra University, Shaqra, Saudi Arabia”

The authors apologize for this error and state that this does not change the scientific conclusions of the article in any way. The original article has been updated.

